# Ghosts From the Past: A Juvenile Onset Huntington's Disease Case From Bahrain

**DOI:** 10.1192/bjo.2024.666

**Published:** 2024-08-01

**Authors:** Hanan Husain, Shaima Bucheeri, Feras Alsaif

**Affiliations:** Governmental Hospitals, Manama, Bahrain

## Abstract

**Aims:**

Huntington’s disease (HD) is a rare inherited disease in an autosomal dominant pattern, that is most prevalent among Caucasians.

Juvenile onset Huntington disease (JHD) is a rare subtype of the disease, defined by presence of the disease by the age of 20 or younger.

We report a case of a 28-year-old woman with JHD and discuss the challenges we faced in her diagnosis and management.

**Methods:**

A now 28-year-old Arab woman, presented to the psychiatric hospital when she was an 18-year-old, complaining of restlessness and low mood. She was diagnosed to be having social phobia and panic attacks, and was given escitalopram. About 6 months after her first presentation, the patient's mother showed up reporting that the patient is doing well without the medication and that she is not going to take them anymore. However, the patient started developing anxiety symptoms two years later and started taking the same medication. Moreover, three years after her first presentation, the patient started developing movement symptoms and mentioned that her father passed away by Huntington's disease. The patient was immediately referred to a genetic lab and a Huntington disease diagnosis was given along with tetrabenazine and risperidone. Moreover, the patient attempted suicide multiple times after worsening of symptoms over the years. A brain magnetic resonance imaging of the patient showed bilateral caudate nuclei atrophy with similar changes affecting the putamen as well but to a lesser extent, changes that are associated with Huntington's disease.

**Results:**

Juvenile onset Huntington disease is rare, especially among people who are not of European ancestry. Therefore, clinicians are not likely to suspect the illness at an early stage. Late diagnosis not only can prevent patients from receiving the symptomatic treatments that they need, but it can also lead to misdiagnosis. Early referral to genetic testing is required among patients presenting with symptoms and a positive family history. However, risk of suicide is high among patients of Huntington's disease.

**Conclusion:**

Juvenile onset Huntington disease is extremely rare. Initial symptoms of the disease could vary and can be misdiagnosed as epilepsy, mood or behavioral disorders, or schizophrenia. Genetic testing is controversial for patients below 18 years old. Having a low suspicion threshold in diagnosing patients with positive family history of HD who are presenting with such symptoms is recommended. There is no cure and treatment is symptomatic.

